# Epicardial Ablation of Focal Atrial Tachycardia Arising From Left Atrial Appendage in Children

**DOI:** 10.1016/s0972-6292(16)30776-8

**Published:** 2014-07-15

**Authors:** Abdhija Hanumandla, Daljeet Kaur, Mandar Shah, Narasimhan Calambur

**Affiliations:** Care Hospital, the Institute of Medical Sciences, Hyderabad, India

**Keywords:** Focal atrial tachycardia, Epicardial Ablation, Children

## Abstract

Focal left atrial tachycardia (FLAT) although a common cause of supraventricular tachycardia(SVT) among children, the one's arising from left atrial appendage (LAA) present a unique challenge for successful ablation because of anatomical location. We present two children with FLAT arising from the epicardial LAA, successfully mapped and ablated through percutaneuous epicardial approach.

## Introduction

The LAA is one of the major sources of FLATs in children [1]. However, due to a large number of trabeculations and true epicardial`location, radiofrequency ablation (RFA) in an atrial appendage may fail or there may be recurrence of FLAT; and surgical appendectomy / cryoablation /video assisted thoracoscopy may be required [2-6]. We report two children with FLAT originating from epicardial LAA with tachycardia induced cardiomyopathy (TIC), in whom RFA was successfully done through epicardial approach without any complications with subsequent improvement in left ventricular ejection fraction.

## Case 1

**Figure 1:**
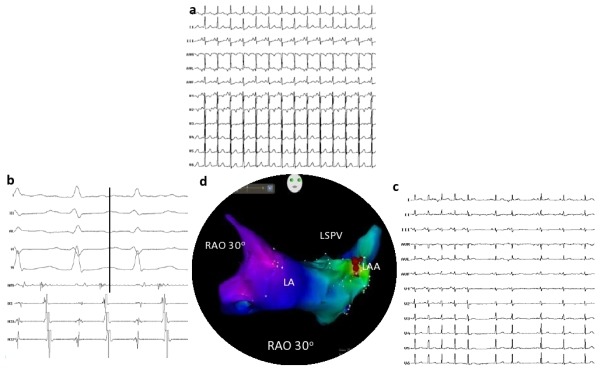
(a) 12 lead ECG of baseline tachycardia, suggestive of high left atrial tachycardia; (b) intracardiac electrograms revealing earliest A at base of left atrial appendage 28 ms ahead of surface P wave; (c) 12 lead ECG of termination of tachycardia during RFA; (d) electroanatomic image of site of ablation at the base of left atrial appendage (RAO 30o); LAA (Left atrial appendage); LA (left atrium); LSPV (Left superior pulmonary vein).

A 12 year old girl presented with history of recurrent episodes of palpitations and fatigue. She had left ventricular dysfunction secondary to (TIC). Tachycardia was refractory to multiple antiarrhythmic drugs and was referred for RFA. Patient was in incessant atrial tachycardia and baseline 12 lead electrocardiogram (ECG) revealed negative P waves in leads I and avL, positive P wave in leads V1, III and avF and isoelectric P wave in lead II (Figure 1). Activation mapping revealed a distal- to- proximal atrial activation sequence in coronary sinus. The diagnosis of atrial tachycardia was confirmed by intermittent AV block with continuation of tachycardia. 3D Carto guided electroanatomic mapping (EAM) of the left atrium was initially performed using antegrade transseptal approach. The earliest A was found to be at the base of the LAA during tachycardia, 30ms ahead of surface QRS. However, at the site of earliest activation, the unipolar signals from the mapping catheter showed a positive deflection. Despite extensive mapping, unipolar signals consistently revealed r waves at the sites of earliest activation. Hence, pericardial puncture done and mapping was performed in the epicardial surface of LAA. The earliest activation during tachycardia was found overlying the base of LAA (28ms ahead of surface P wave, this time with steep QS pattern of unipolar signals, Figure 1). This site was adjacent to the endocardial site of earliest activation. Radiofrequency application at this epicardial site with 7.5F irrigated tip catheter eliminated the tachycardia within 5 seconds of onset of energy. The patient has now completed 7 months of follow-up, without any recurrence of tachycardia. The left ventricular function has improved to normal.

## Case 2

**Figure 2:**
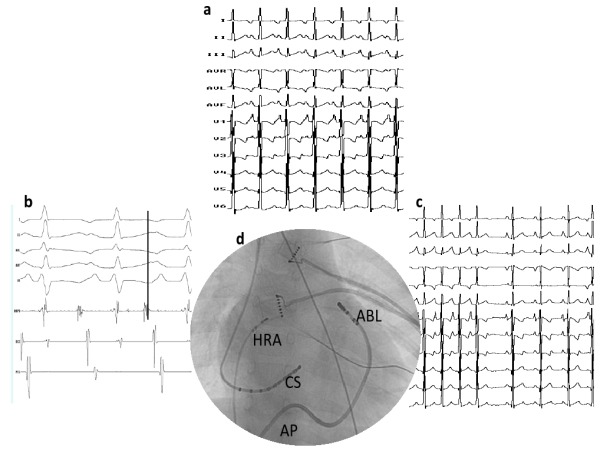
(a) 12 lead ECG of baseline tachycardia, suggestive of high left atrial tachycardia; (b) intracardiac electrograms revealing earliest A at base of left atrial appendage 47 ms ahead of surface P wave; (c) termination of tachycardia during RFA; (d) site of ablation at the base of left atrial appendage; HRA (high right atrium); ABL (ablation catheter); CS (coronary sinus); AP (anterior posterior)

The second case was a 6 year old boy presented to us with incessant atrial tachycardia at the rate of 190 per minute with 1:1 AV conduction and TIC. The 12 lead ECG showed negative P waves in leads I and avL and positive P waves in the inferior leads and in lead V1 (Figure 2). He had undergone RFA endocardially twice in the past using 3D EAM guidance, and on both the occasions, the tachycardia was terminated by radiofrequency energy in the base of LAA. However, he had recurrence on both the occasions. Hence this time he was taken for elective epicardial ablation. Pericardial puncture was done and mapping was performed directly in the epicardial surface of LAA under 3D NavX guidance. Mapping revealed the earliest activation in the base of LAA, 47ms ahead of surface QRS, with early QS in Unipolar signals (Figure 2). Radiofrequency energy at this site with non irrigated tip catheter terminated tachycardia soon after starting the energy without any complications. This patient has now completed two years of follow-up and is free of atrial arrhythmia without any antiarrhythmic drugs with improvement in cardiac function.

## Discussion

There have been several case series of successful catheter ablation of an LAA tachycardia using different approaches [1-6]. To the best of our knowledge, this is the first report on two children illustrating a FLAT with an epicardial LAA origin that was successfully ablated through percutaneous epicardial approach. Although, previously few cases have been reported with similar approach in adults and adolescents [7,8] , none have been reported in children. Catheter ablation using an epicardial access by a pericardial puncture may be necessary and is a proven feasible route for the management of a variety of arrhythmias especially the ventricular tachycardias [9]. On the other hand atrial arrhythmias are generally amenable to endocardial catheter ablation because a transmural lesion can be made in the thinner atrial myocardial wall. Failure of endocardial approach to ablate the atrial tachycardia origin in our cases might be attributed to the true epicardial locations and the thick pectinate muscle within the LAA.

Thus, the epicardial catheter ablation may be an alternative approach to endocardial catheter ablation in atrial tachycardias with an LAA origin without the need for subjecting these patients to surgical ablation.
